# One year soy protein supplementation has positive effects on bone formation markers but not bone density in postmenopausal women

**DOI:** 10.1186/1475-2891-4-8

**Published:** 2005-02-23

**Authors:** Bahram H Arjmandi, Edralin A Lucas, Dania A Khalil, Latha Devareddy, Brenda J Smith, Jennifer McDonald, Andrea B Arquitt, Mark E Payton, Claudia Mason

**Affiliations:** 1 Department of Nutritional Sciences, Oklahoma State University, Stillwater, Oklahoma 74078, USA; 2 Department of Statistics, Oklahoma State University, Stillwater, Oklahoma 74078, USA

## Abstract

**Background:**

Although soy protein and its isoflavones have been reported to reduce the risk of osteoporosis in peri- and post-menopausal women, most of these studies are of short duration (i.e. six months). The objective of this study was to examine if one year consumption of soy-containing foods (providing 25 g protein and 60 mg isoflavones) exerts beneficial effects on bone in postmenopausal women.

**Methods:**

Eighty-seven eligible postmenopausal women were randomly assigned to consume soy or control foods daily for one year. Bone mineral density (BMD) and bone mineral content (BMC) of the whole body, lumbar (L1-L4), and total hip were measured using dual energy x-ray absorptiometry at baseline and after one year. Blood and urine markers of bone metabolism were also assessed.

**Results and Discussion:**

Sixty-two subjects completed the one-year long study. Whole body and lumbar BMD and BMC were significantly decreased in both the soy and control groups. However, there were no significant changes in total hip BMD and BMC irrespective of treatment. Both treatments positively affected markers of bone formation as indicated by increased serum bone-specific alkaline phosphatase (BSAP) activity, insulin-like growth factor-I (IGF-I), and osteocalcin (BSAP: 27.8 and 25.8%, IGF-I: 12.8 and 26.3%, osteocalcin: 95.2 and 103.4% for control and soy groups, respectively). Neither of the protein supplements had any effect on urinary deoxypyridinoline excretion, a marker of bone resorption.

**Conclusion:**

Our findings suggest that although one year supplementation of 25 g protein per se positively modulated markers of bone formation, this amount of protein was unable to prevent lumbar and whole body bone loss in postmenopausal women.

## Background

It is estimated by the year 2010, 35 million women in the United States either will have osteoporosis or be at risk of developing the disease if appropriate preventive measures are not taken [1]. Aside from existing drug therapies, certain lifestyle and nutritional factors are known to reduce the risk of osteoporosis [2-5]. Additionally, there are a considerable number of women that would prefer dietary supplements as an alternative/adjunctive to conventional therapeutic options [5]. Examples of these alternative therapies include the use of natural or plant-based substances such as soy isoflavones [6-13]. Soy isoflavones have received considerable attention due to their estrogen-like properties on certain tissues such as bone, leading some investigators [14,15] to refer to them as naturally occurring selective estrogen receptor modulators (SERMs).

Epidemiological data suggest that populations with high intakes of soy, i.e. Asians, have a lower incidence of osteoporotic fractures [16,17]. Asian women typically consume about 20 g of soy daily which provides approximately 40 mg isoflavones [18,19]. However, lower rates of fractures in these populations may not be fully attributed to soy consumption as there are a number of other confounding factors which can influence skeletal health.

From the research point of view, there are a number of animal studies which have shown that soy protein and/or its isoflavones positively influence bone mineral density (BMD) [8,20-26]. In terms of human studies, there are limited numbers of trials that have examined the effects of soy and its isoflavones on bone. Some of these clinical trials [27-29] are of short duration varying between 3 to 6 months, making the findings questionable since periods less than one year may not be sufficient to detect clinically relevant changes in bone mass. Nonetheless, even the findings of the few clinical studies of one to two year duration that have been conducted [30-34] are inconclusive. For instance, Vitolins et al. [30] reported that daily consumption of 25 g soy protein with 5, 42 or 58 mg isoflavones had no bone preserving effects in peri- and post-menopausal women in a two-year study. Recent findings from two groups [31,32] have shown similar effects of soy protein and its isoflavones on bone. In contrast, other studies have suggested that isoflavone-rich soy milk delivering 80 to 90 mg isoflavones [33] or soy products delivering 40 to 60 mg isoflavones on a daily basis [34] have some bone protective effects.

The purpose of the present study was to examine the effects of one-year supplementation of soy-based products containing 25 g protein and 60 mg isoflavones on BMD, bone mineral content (BMC), serum and urinary markers of bone turnover in postmenopausal women. The rationale for choosing this amount of soy protein was based on the average intake of Asians [18,19] and the recommended amount in the FDA approved health claim [35].

## Methods

### Subjects

Postmenopausal women younger than 65 years who were not on HRT or any prescription medications or herbal supplements, including soy isoflavones, known to positively influence bone were recruited. Women with cancer, liver disease, hypo- or hyperthyroidism, gastrointestinal disorders, insulin-dependent diabetes mellitus, pelvic inflammatory disease, and endometrial polyps were excluded from the study. The study protocol was approved by the Institutional Review Board at Oklahoma State University. Subjects signed a consent form after being provided with oral and written descriptions of the study. A complete medical history was obtained from all subjects before initiating the treatments. Subjects were also given routine physical and gynecological examinations. Subjects were independent living and were advised to maintain their usual physical activity.

### Study Design

Eighty-seven eligible postmenopausal women were randomly assigned to one of two dietary treatments in a double-blind parallel study. The dietary treatments consisted of 25 g protein from soy products (donated by DrSoy Nutrition Irvine, CA) or comparative control. The test foods were in the form of a snack bar, drink mix or cereal and were consumed daily for a period of one year. The soy products were soy protein-based and delivered 60 mg isoflavones per day whereas the control regimen was devoid of soy protein and isoflavones.

To ensure double-blinding, the study participants were randomly assigned to one of the two treatments and the study supplies were provided to the study participants in unlabeled packages. Additionally, the identity of each treatment was revealed to the investigators and research personnel involved in the collection and analyses of the data only after all analyses were completed. For this study compliance was measured in two forms. First, study participants were provided with customized calendars for subjects to record how much of each of the cereal, the snack bar, or the drink mix they consumed, if any. Second, study participants were asked to return any unconsumed foods to the study site so they could be tallied. The study participants were advised by a registered dietitian to make appropriate adjustments in their daily food consumption to account for the additional energy and nutrients supplied by the treatment regimen.

### Dietary assessment and anthropometric measurements

For each subject, medical and nutrition histories were obtained at the beginning of the study. One-week food frequency questionnaires were completed via interview by a registered dietitian at the beginning and at the end of the study. Nutrient analysis was performed using food analysis software (Food Processor version 7.50, ESHA Research, Salem, OR). Anthropometric data were collected at the beginning, six months, and at the end of the study by a single trained staff member, as described elsewhere [36]. Height and weight were used to calculate body mass index (BMI). Abdominal and hip circumferences were used to calculate waist-to-hip ratio.

### Bone Density Assessments

Bone density was assessed at the beginning and at the end of treatment using dual energy x-ray absorptiometry (DXA; Hologic QDR-4500C, Waltham, MA) equipped with appropriate software for whole body BMD and BMC. Additionally, select regional sites, i.e. total hip and lumbar spine (L1-L4) were analyzed using high resolution software. The intra- and inter- assay coefficients of variations were 3.4% and 5.1% and 2.5% and 4.7% for BMC and BMD, respectively.

### Gynecological exam and blood and urine collection

Study participants were provided with routine physical and gynecological exams including a pap smear at baseline and at the end of the study. A venous blood sample was obtained after an overnight fast from each subject at the beginning, six months, and at the end of the study for various analyses. Blood samples were centrifuged at 2500 × g for 15 min at 4°C, serum samples were separated and stored at -20°C until analyses. Each study participant collected a 24-h urine specimen, excluding the first void, at the beginning, after six months, and at the end of the study. Urine volume was recorded and aliquots were stored at -20°C for later analyses.

### Analytical methods

To assess whether soy isoflavones modulate sex steroids and their availability, serum levels of 17β-estradiol (E_2_), estrone (E_1_), estrone sulfate, follicle stimulating hormone (FSH), and sex hormone-binding globulin (SHBG) were assessed as we have previously described [37] using radioimmunoassay kits from Diagnostic Systems Laboratories Inc. (Webster, TX). Serum bone-specific alkaline phosphatase (BSAP) activity, a specific marker of bone formation [38], was quantified by immunoassay in a microtiter format (Metra Biosystems, Mountain View, CA). Alkaline phosphatase (ALP), a nonspecific marker of bone formation [38], was determined colorimetrically using a commercially available kit (Roche Diagnostics; Branchburg, NJ) and analyzed with a Cobas-Fara II Clinical Analyzer (Montclair, NJ). Additional serum biomarkers of bone formation i.e. osteocalcin, insulin-like growth factor-I (IGF-I), and IGF-binding protein-3 (IGFBP-3), which usually increases parallel to IGF-I, were also measured using kits from Diagnostic Systems Laboratories Inc.

Urinary creatinine was measured colorimetrically with commercially available kits (Roche Diagnostics) using a Cobas Fara II clinical analyzer. Urinary deoxypyridinoline (Dpd), a specific marker of bone resorption [39], was measured by competitive enzyme immunoassay in a microassay stripwell format (Quidel Corporation, Mountain View, CA). The intra- and inter-assay CVs were 4.3% and 4.6 %, and 6.5% and 8.6%, for creatinine and Dpd, respectively.

### Statistical Analyses

Data were analyzed using analysis of variance methods with PROC MIXED in PC SAS (Version 8.2, SAS Institute, Cary, NC) analyzing the main and interaction effects of the two factors, treatment (soy protein or control) and time (baseline or after treatment), using the SLICE option. Since each subject was measured at baseline and after treatment, a split plot (repeated measures) model was utilized. The mean changes in endpoints for the soy protein and control treatment groups were compared by analyzing interaction effects of the two factors, treatment and time, using the SLICE option. Data are reported as least square mean ± standard error (SE); unless otherwise indicated, *P *< 0.05 was regarded as significant.

## Results

### Baseline characteristics, anthropometric measurements, and dietary intake

Sixty-two of the 87 women completed the one year study resulting in an attrition rate of approximately 29%. Reasons for dropping from the study included medical conditions preventing continuation in the study (2 women in the soy group and 1 in the control group), starting HRT (1 woman in the soy group and 3 in the control group), noncompliance (2 women in the soy group), dislike of the volume or flavor of the food (3 women in the control group), gastrointestinal side effects (2 women in the control group), food was causing headaches (1 woman in the control group), and personal reasons (3 women in the soy group and 2 in the control group). Five additional women in the soy group decided to discontinue the study without citing a particular reason.

Women in both treatment groups had similar baseline characteristics (Table 1). Body weight and BMI significantly increased in both treatment groups after one year of supplementation. On average, women in the soy group experienced a 1.6% increase in body weight, while a 3.3% increase was observed in women in the control group. Nonetheless, there was no significant change in waist to hip ratio in either treatment group. In terms of dietary intake, protein levels significantly increased in both treatment groups, as expected, due to the study supplements. Total caloric and carbohydrate intake increased in the control group, but decreased in the soy group after one year of supplementation (Table 2). No change in fat intake was found in either group.

### Bone mineral density and bone mineral content

**Figure 1:**
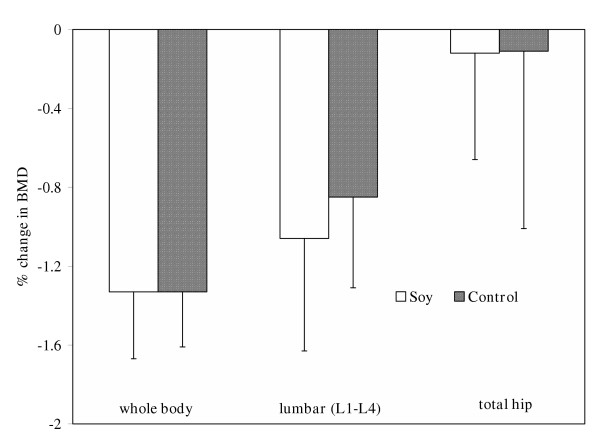
Mean percent change from baseline values in bone mineral density (BMD) of the whole body, lumbar (L1-L4), and hip after one year of supplementation with control or soy foods. Bars represent least square means ± SE.

**Figure 2:**
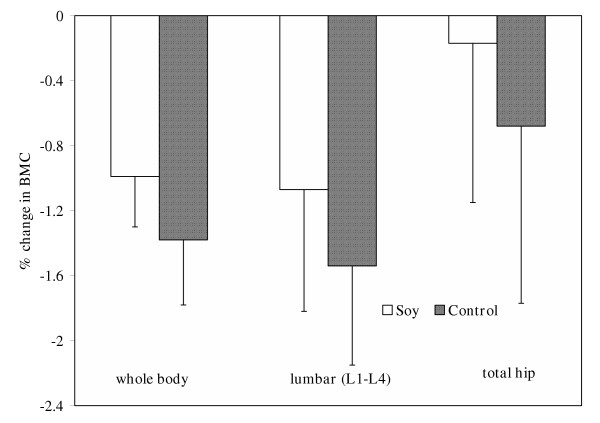
Mean percent change from baseline values in bone mineral content (BMC) of the whole body, lumbar (L1-L4), and hip after one year of supplementation with control or soy foods. Bars represent least square means ± SE.

Subjects in both the soy and control groups lost whole body and lumbar BMD and BMC after one year, but no change was observed in the total hip (Table 3). Whole body BMD decreased by approximately 1.3% in both treatment groups, while lumbar BMD decreased by 1.0% in the soy group and 0.9% in controls (Figure 1). As shown in Figure 2, whole body BMC decreased by 1.4% and 1.0%, and lumbar BMC was reduced by 1.5% and 1.1% in the control and soy groups, respectively. Although the change in whole body bone mineral area (BMA) of the soy-treated group was increased and the control group remained relatively unchanged, neither of these alterations reached the level of statistical significance (data not shown). The decrease in whole body BMD of subjects on soy may have resulted from the small increase in whole body area and a significant decrease in BMC, whereas the change in BMD in the control group was primarily a reflection of a decrease in BMC. Lumbar and total hip BMA was unaltered by either dietary treatment (*data not shown)*.

### Serum and urinary parameters of relevance to bone and calcium metabolism

Both dietary protein supplements significantly increased serum markers of bone formation, i.e. osteocalcin, BSAP, IGF-I and ALP (Table 4). However, IGFBP-3, which usually increases parallel to IGF-I, was only significantly increased in the women consuming the soy products. Neither proteins had any effect on bone resorption as indicated by urinary Dpd (Table 4).

Since there are some reports [40-42] indicating that soy isoflavones may modulate sex steroids, we assessed serum levels of FSH, E_2_, E_1_, and estrone sulfate. None of the treatment significantly influenced these sex hormones (Table 5). However, soy but not control supplementation significantly decreased SHBG concentrations by 14.5%.

## Discussion

The role of soy protein and its isoflavones in the maintenance of health such as the prevention of cardiovascular disease, certain types of cancer, and menopausal symptoms is now widely recognized [43-50]. In terms of bone, there are animal [20-26] and human [27-34] studies that have explored the role of soy in maintaining or increasing bone mass. In general, animal studies have shown that isoflavones in the context of soy protein have positive effects on BMD [20-26]. The findings of clinical trials have ranged from no significant changes [27-32] or a slight increase [28,33,34] in BMD. Nonetheless, the bone protective effects of soy and/or its isoflavones are at best inconclusive.

In the present study, the daily consumption of 25 g protein for one year irrespective of the source resulted in no significant changes in hip BMD and BMC. Other investigators [51,52] have reported that diets high in protein were associated with higher BMD in femoral neck. We speculate that higher dietary protein may have a protective effect on hip BMD over the long term. This notion, however, seems somewhat paradoxical because high protein diets, especially proteins rich in sulfur-containing amino acids, are known to increase urinary calcium that may result in accelerated bone loss [53]. Nonetheless, a counter-argument has been made that protein-associated hypercalciuria is due to enhanced intestinal calcium absorption and not the breakdown of bone [54,55].

Our findings do not support a bone protective role for soy protein and its isoflavones at the level used in this study. Whether higher amount of soy protein and/or its isoflavones can reverse bone loss remains to be illustrated. Nonetheless, higher doses of soy protein with varying levels of isoflavones have not consistently shown to exert beneficial effects on bone. For instance, Gallagher et al. [32] supplemented the diets of postmenopausal women for nine months with 40 g soy protein delivering three levels of isoflavones (0, 52, and 96 mg) but all three groups experienced bone loss. On the other hand, six-month studies by Potter et al. [28] and Alekel and colleagues [29] reported positive effects of soy protein supplementation on BMD. Potter et al. [28] showed that 40 g of soy protein containing 90 mg isoflavones was able to attenuate lumbar spine (L1-L4) BMD, however, the same amount of protein with 56 mg isoflavones had no such an effect. Although Alekel et al. [29] suggested that 40 g soy protein supplementation with 80 mg isoflavones was able to attenuate bone loss from lumbar spine, women still lost 0.2% BMD in six months. Their data [29] imply that soy protein or its isoflavones are incapable of increasing bone mass in perimenopausal women.

As for an effect of soy isoflavones alone, Chen et al. [56] recently reported that supplementing postmenopausal women with soy isoflavones (40 and 80 mg/d) for one year resulted in favorable increases in BMC of the hip in women who are at least four years postmenopausal and are of low body weight or have low levels of dietary calcium. Similar to that study [56], the majority of the women in our present study were four or more years postmenopausal; however, our study participants had adequate calcium intakes and did not have low body weights. It is possible that this difference in the nutrition status of the study participants between the two studies may be responsible for the discrepancy in the observed effects on bone.

As far as which of the many isoflavones in soy is responsible for the effects on bone, to date, the most convincing data on the effect of a single isoflavone, genistein, on bone have been reported in a one-year study by Morabito and colleagues [57]. They demonstrated that both genistein at a dose of 54 mg/d and HRT increased BMD in early postmenopausal women. In that study [57], genistein significantly increased BMD of the femoral neck by 3.6% and lumbar spine by 3.0% while HRT increased femoral neck and lumbar spine BMD by 2.4 and 3.8%, respectively. These authors [57] suggested that genistein reduces bone resorption markers and enhances new bone formation parameters resulting in a net gain of bone mass. Isolated isoflavones derived from other sources such as red clover have also been found to positively affect bone. For example, Clifton-Bligh and colleagues [58] reported that clover-derived isoflavones at doses of 57 and 85 mg isoflavones/day were able to significantly increase BMD of the proximal radius and ulna by 4.1 and 3%, respectively after 6 months. Similarly, Atkinson et al. [59] showed that red-clover derived isoflavones (26 mg biochanin A, 16 mg formononetin, 1 mg genistein, and 0.5 mg daidzein) slowed the loss of lumbar spine BMC and BMD. These data suggest that isoflavones from sources other than soy, also have osteoprotective effects.

In the present study, biomarkers of bone formation, i.e. osteocalcein, IGF-I, and BSAP were all significantly elevated in both groups. However, the specific marker of bone resorption, urinary Dpd, was not altered. A number of clinical studies have evaluated the effects of soy protein with its isoflavones on bone biomarkers. Overall, the effects of soy and/or its isoflavones have produced no consistent effects on biomarkers of bone turnover. For instance, biomarkers of bone formation have been reported to either increase [60,61] or not change [29,62] as result of soy supplementation. Similarly, biomarkers of bone resorption have been reported to decrease [62-64], not change [61] or even increase [60]. We have previously reported that 40 g of soy protein providing 90 mg of isoflavones/day reduced Dpd in postmenopausal women not on HRT [62]. In the present study, participants were asked to consume a lower amount of soy protein (only 25 g providing 60 mg of isoflavones/day). It is possible that the reduced dose of soy in this study may have contributed to the lack of effect on bone resorption (as assessed by Dpd excretion) and that a dose-response study may be justified in order to achieve both increases in markers of bone formation and reductions in markers of bone resorption. Nevertheless, the positive changes in biomarkers of bone formation in the present study have not translated to increases in BMD and BMC. Whether the positive effects of protein supplementation on bone biomarkers would translate to better bones needs to be assessed in a longer term study.

Although in this study, soy supplementation for one year did not produce any estrogenic effects as assessed by circulating sex hormone levels, it did decrease SHBG concentrations Decreases in SHBG result in increases in the availability of circulating estrogens [65]. Thus, soy supplementation may have increased the availability of estrogens without affecting actual concentrations. However, this is speculative and measurement of bioavailable estradiol is necessary to confirm this statement.

From the findings of our study and the collective review of existing literature, it is too early to state whether soy protein or its isoflavones can be substituted for estrogen in preventing the bone loss induced by ovarian hormone deficiency. Future studies are needed to address numerous questions including but not limited to whether: 1) isoflavones independent of soy protein can prevent ovarian hormone deficiency-associated bone loss; 2) consumption of soy containing food or intake of isoflavones on a daily basis is necessary to observe the expected beneficial effects on bone or simply intermittent use will produce the same results; 3) the effect of soy protein or its isoflavones on bone is transitory; and 4) the combination of soy isoflavones and lower doses of antiresorptive agents can prevent postmenopausal bone mineral loss. As these and other questions are answered, the efficacy of soy protein and its isoflavones as alternative and/or adjunctive treatments for postmenopausal osteoporosis can be determined.

## Competing interests

The author(s) declare that they have no competing interests.

## Figures and Tables

**Table 1:** Subject characteristics at baseline and at the end of the study

**Measures**	**Control **(n = 27)			**Soy **(n = 35)			**Control vs Soy**		
	Baseline	Final	%change	Baseline	Final	%change	*p *values		
							Trtmt	Time	Trtmt × Time
Age (*yrs*)	56 ± 5			53 ± 6					
Years since menopause	6 ± 5			5 ± 5					
Weight *(kg)*	71.7 ± 2.4	74.1 ± 2.4	+3.3	75.5 ± 2.2	76.7 ± 2.2	+1.6	0.334	<0.001	0.170
BMI *(kg/m^2^)*	27.3 ± 1.0	28.2 ± 1.0	+3.3	28.6 ± 0.9	29.0 ± 0.9	+1.4	0.436	<0.001	0.178
Waist to hip ratio	0.817 ± 0.014	0.815 ± 0.014	-0.2	0.787 ± 0.013	0.788 ± 0.013	+0.1	0.114	0.921	0.827

Values are least squares mean ± SE. BMI = body mass index.

No treatment effects were detected.

**Table 2:** Daily total energy and macronutrient intake at baseline and after one year supplementation of soy or control foods

Measures	**Control **(n = 27)		**Soy **(n = 35)		**Control vs Soy**		
	Baseline	Final	Baseline	Final	*p *values		
					Trtmt	Time	Trtmt × Time
Total energy *(kcal)*	1577 ± 95^b^	1850 ± 96^a^	1827 ± 82^a^	1582 ± 84^b^	0.933	0.853	0.001
Protein *(g)*	64.2 ± 4.1^c^	87.8 ± 4.1^a^	75.8 ± 3.6^b^	87.3 ± 3.6^a^	0.241	<0.001	0.035
Carbohydrates *(g)*	207 ± 14^bc^	247 ± 14^a^	243 ± 12^ab^	202 ± 12^c^	0.752	0.938	<0.001
Total fat *(g)*	56.6 ± 4.8	59.0 ± 4.8	62.5 ± 4.1	57.0 ± 4.2	0.719	0.667	0.277
Calcium *(mg)*	796 ± 69	1168 ± 70	873 ± 60	1183 ± 62	0.459	0.003	0.777

Values are least squares mean ± SE.

Values were calculated from 7-day food frequency questionnaires and included the treatment regimen.

Within each row, values that do not share the same superscript letters are significantly (*P *< 0.05) different from each other. Soy food products on the average provided approximately 232 kcal, 25 g protein, 4.4 g fat, 25 g carbohydrates, 500 mg calcium and 60 mg isoflavones daily. Control food products on the average provided approximately 454 kcal, 25 g protein, 6 g fat, 77 g carbohydrates, 500 mg calcium and 0 mg isoflavones daily. Soy and control foods were donated by DrSoy Nutrition (Irvine, CA).

**Table 3:** Effects of control and soy foods on bone mineral density (BMD) and bone mineral content (BMC)

**Measures**	**Control **(n = 27)		**Soy **(n = 35)		**Control vs Soy**		
	Baseline	Final	Baseline	Final	*p *values		
					Trtmt	Time	Trtmt × Time
**BMD, ***(g/cm^2^)*							
Whole body	1.050 ± 0.020	1.036 ± 0.0201	1.050 ± 0.018	1.036 ± 0.018	0.986	< 0.001	0.936
L1-L4	0.941 ± 0.026	0.933 ± 0.026	0.944 ± 0.022	0.934 ± 0.022	0.958	0.039	0.825
Hip, total	0.871 ± 0.021	0.870 ± 0.021	0.853 ± 0.018	0.852 ± 0.018	0.512	0.904	0.988
**BMC, ***(g)*							
Whole body	2022 ± 58	1994 ± 58	2023 ± 49	2003 ± 49	0.944	< 0.001	0.465
L1-L4	53.631 ± 2.137	52.806 ± 2.137	54.737 ± 1.816	54.152 ± 1.816	0.662	0.018	0.681
Hip, total	57.532 ± 2.051	57.140 ± 2.051	57.278 ± 1.743	57.182 ± 1.743	0.967	0.738	0.839

Values are least squares mean ± SE. Lumbar spine (L1-L4).

No treatment effects were detected.

**Table 4:** Effects of control and soy foods on serum and urinary markers of bone metabolism

**Measures**	**Control **(n = 27)			**Soy **(n = 35)			**Control vs Soy**		
	Baseline	6 months	Final	Baseline	6 months	Final	*P *values		
							Trtmt	Time	Trtmt × Time
***Serum***									

IGF-I *(nmol/L)*	17.9 ± 1.4^b^	20.5 ± 1.4^a^	20.2 ± 1.4^a^	13.3 ± 1.2^c^	16.8 ± 1.2^ab^	16.8 ± 1.2^ab^	0.023	< 0.001	0.693
IGFBP3 *(ng/mL)*	3833 ± 133	3934 ± 133	3904 ± 135	3442 ± 120^b^	3751 ± 120^a^	3622 ± 121^a^	0.087	0.005	0.252
Osteocalcin *(ng/mL)*	8.3 ± 2.1^b^	13.9 ± 2.1^a^	16.2 ± 2.2^a^	8.9 ± 1.9^b^	17.4 ± 2.0^a^	18.1 ± 2.0^a^	0.365	< 0.001	0.671
BSAP *(U/L)*	19.8 ± 1.2^c^	23.3 ± 1.2^b^	25.3 ± 1.2^a^	19.8 ± 1.1^c^	22.6 ± 1.1^b^	24.9 ± 1.1^a^	0.796	< 0.001	0.818
ALP *(U/L)*	57.4 ± 3.0^c^	65.9 ± 3.0^b^	74.0 ± 3.1^a^	56.6 ± 2.7^c^	69.1 ± 2.7^b^	74.3 ± 2.8^a^	0.798	< 0.001	0.499
***Urine***									
Dpd *(nmol/mmol creatinine)*	5.3 ± 0.4	5.3 ± 0.4	4.9 ± 0.4	5.2 ± 0.3	5.4 ± 0.3	5.1 ± 0.3	0.888	0.234	0.744

Values are least squares mean ± SE. IGF-I = insulin-like growth factor-I; IGFBP-3 = insulin-like growth factor binding protein-3; BSAP = bone-specific alkaline phosphatase; ALP = alkaline phosphatase; Dpd = deoxypyridinoline.

Within each row, values that do not share the same superscript letters are significantly (*P *< 0.05) different from each other.

**Table 5:** Effects of control and soy foods on on sex hormones

**Measures**	**Control **(n = 27)			**Soy **(n = 35)			**Control vs Soy**		
	Baseline	6 months	Final	Baseline	6 months	Final	*P *values		
							Trtmt	Time	Trtmt × Time
FSH *(mIU/mL)*	41.0 ± 3.8	37.5 ± 3.8	36.4 ± 3.8	44.8 ± 3.4	47.6 ± 3.4	43.2 ± 3.4	0.143	0.195	0.235
E_2 _*(pg/mL)*	9.9 ± 4.1	12.3 ± 4.1	8.8 ± 4.2	12.7 ± 3.7	19.5 ± 3.7	17.7 ± 3.8	0.120	0.314	0.690
Estrone *(pg/mL)*	14.3 ± 4.3	21.9 ± 4.3	13.9 ± 4.4	19.6 ± 3.8	22.0 ± 3.9	22.4 ± 3.9	0.283	0.290	0.448
Estrone sulfate *(ng/mL)*	1.2 ± 0.4	1.9 ± 0.4	1.1 ± 0.4	1.8 ± 0.4	2.0 ± 0.4	1.9 ± 0.4	0.264	0.309	0.552
SHBG *(nmol/L)*	87.1 ± 9.2	81.3 ± 9.2	76.3 ± 9.3	105.3 ± 8.2	88.2 ± 8.2	90.0 ± 8.3	0.264	0.002	0.355

Values are least squares mean ± SE. FSH = follicle stimulating hormone; E_2 _= 17β-estradiol; SHBG = sex hormone-binding globulin.

No treatment effects were detected.
